# Effect of Gradient Transition Layer on the Cracking Behavior of Ni60B (NiCrBSi) Coatings by Laser Cladding

**DOI:** 10.3390/ma18020419

**Published:** 2025-01-17

**Authors:** Qi Sun, Weiming Bi, Shan Yao, Wenxu Zhu, Wenjian Ma, Bing Hu, Cuimin Bao, Yong Zhang, Fangyong Niu

**Affiliations:** 1 School of Materials Science and Engineering, Dalian University of Technology, Dalian 116024, China; sunqi43793870@163.com; 2 Shengu Group Co., Ltd., Shenyang 110869, China; mawenjian@shengu.com.cn (W.M.); hubing712@163.com (B.H.); baocm@hotmail.com (C.B.); zhangy@shengu.com.cn (Y.Z.); 3 State Key Laboratory of High-Performance Precision Manufacturing, Dalian University of Technology, Dalian 116024, China; weimingbi@163.com; 4 Xiaomi Communications Co., Ltd., Beijing 100085, China; zhu5977@126.com

**Keywords:** laser cladding, Ni60B, transition layer, cracking inhibition

## Abstract

Laser cladding technology is an effective method for producing wear-resistant coatings on damaged substrates, improving both wear and corrosion resistance, which extends the service life of components. However, the fabrication of hard and brittle materials is highly susceptible to the problem of cracking. Using gradient transition layers is an effective strategy to mitigate the challenge of achieving crack-free laser-melted wear-resistant coatings. This study presents the cracking issue of laser cladding Ni60B (NiCrBSi) coatings on 38CrMoAl (18CrNiMo7-6) steel by designing a gradient transition layer infused with varying amounts of Ni powder. We examine how different levels of Ni doping in the transition layer influence the fabrication of the Ni60B coating. The results indicate that the cracking mechanism of Ni60B is primarily due to the brittleness and hardness of the fusion cladding layer, which can result in cold cracks under residual tensile stress. Increasing the nickel content in the transition layer reduces the difference in thermal expansion coefficients between the cladding layer and the substrate. Additionally, the nickel in the transition layer permeates the cladding layer due to the laser remelting effect. The physical phase within the cladding layer transitions from the initial CrB, M_7_C_3_, and γ-Ni solid solution to γ-Ni solid solution and Ni-B-Si eutectic, with a small amount of boride and carbide hard phases. As the nickel doping in the transition layer increases, the proportion of the toughness phase dominated by Ni elements significantly rises, leading to a decrease in the hardness of the fused cladding layer. However, the average hardness of the fusion cladding layer in crack-free samples was measured at 397.5 ± 5.7 HV_0.2_, which is 91% higher than that of the substrate.

## 1. Introduction

The failure of high-value engineering components due to surface damage not only results in the significant waste of raw materials and resources associated with new product purchases and end-of-life inventory but also presents considerable challenges for green development and carbon emission control [1]. Strengthening and repairing the vulnerable surfaces of these components can enhance their comprehensive performance, including corrosion resistance, wear resistance, and fatigue resistance, thereby meeting the performance requirements under various working conditions [2,3,4].

Nowadays, common surface modification technologies include electroplating [5], thermal spraying [6], arc cladding [7], and laser cladding [8]. Among these, laser cladding technology has received significant attention from researchers due to its advantages, such as concentrated heat input, excellent shaping controllability, lower dilution rates, minimal impact on the substrate microstructure, and a wide range of applicable material systems [8,9,10]. However, the high energy density of the laser generates a large temperature gradient in the manufactured sample, leading to significant internal stresses due to the rapid cooling rates. This makes materials like Ni60B, a high-hardness nickel-based self-fusing alloy, particularly susceptible to cracking, which limits the broader adoption of this technology [9,11].

Ni60B is a typical NiCrBSi material that has demonstrated significant potential in the fields of repair and free-form manufacturing [12]. The presence of Cr and C elements in the coatings enhances the formation of hard phases, which significantly improves the microhardness and wear resistance of the samples. However, the issue of cold cracking caused by tensile stress remains a challenge [13]. To address cracking, further doping with Ni in nickel-based alloy powders has been found to mitigate the cracking phenomenon in the fusion cladding layer [14]. Researchers have also adjusted the physical phase composition of the hard phase in the fusion cladding by doping with metals and their oxides, such as Ta, Ti, V, and Nb, achieving improved resistance to cracking [15,16,17,18,19]. Additionally, the introduction of rare-earth elements like Ce, Y, and La, along with their oxides, can refine grain size and enhance the composition of grain boundary elements, potentially reducing defects such as porosity and cracks in the fused cladding layer [20]. While doping elemental materials may improve cladding layer cracking, it can also inadvertently compromise layer performance. Some researchers indicate that the eutectic microstructure formed after toughness element doping may remain brittle and hard, thus limiting the effectiveness of crack inhibition by toughness element [21]. In contrast, the incorporation of a transition layer effectively reduces coating stress and inhibits cracking while also addressing substrate dilution effects [22]. For instance, Tekaya demonstrated that TiN coatings with added Ti/TiN transition layers are effective in reducing stress concentrations [23]. Luo highlighted the beneficial effects of metal–ceramic transition zones on crack suppression [24]. Additionally, Wu showed that transition layers can effectively dissipate crack extension energy, thereby further inhibiting cracking [25].

Up to now, there has been limited research on the development of wear-resistant coatings from the NiCrBSi material system directly applied to nitride steel. In this study, we investigate the use of Ni60B alloy powder as the fusion cladding material, which is known to be susceptible to cracking. To address this issue, we introduce a transition layer consisting of Ni and Ni60B (which includes pure Ni) to inhibit cracking in the fusion cladding layer. We examine the effects of various process parameters and different transition schemes on the crack inhibition of Ni60B coatings, analyzing their microstructure and microhardness in the process. This method not only ensures efficient fabrication and the production of thicker coatings but also enables the achievement of large-area, crack-free cladding. The application of laser cladding technology can be strongly extended to provide guidance in the field of the 3D repair of molds.

## 2. Materials and Methods

### 2.1. Experimental Equipment and Materials

**Figure 1:**
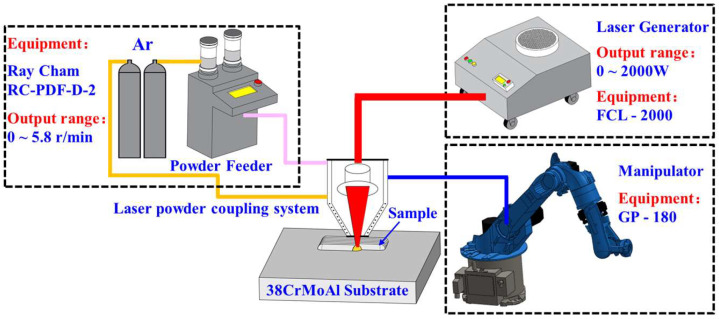
Schematic illustrations of laser cladding forming system.

The fabrication experiments were carried out using a self-constructed laser cladding equipment platform and the composition of the experimental equipment is shown in Figure 1 below. The laser cladding platform consists of a FCL2000 fiber-coupled semiconductor laser (Jilin Changguang Raycus Laser Co., Changchun, China), AK190-SERIES 6KW coaxial powder feeding and deposition equipment (Shanghai Jiaqiang Automation Co., Ltd., Shanghai, China), a GP180 six-axis vertical multi-jointed robot (YASKAWA, Beijing, China), an RC-PGF powder feeder (Nanjing Raycham Laser Technology Co., Nanjing, China), and other equipment. The maximum output power of the laser equipment is 2 kW, the laser wavelength is 1080 ± 5 nm, and the spot size is 3 mm. Notably, 99.99% high-purity argon is used in the experiments to provide atmospheric protection for the molten pool and to transport the ceramic powder.

**Figure 2:**
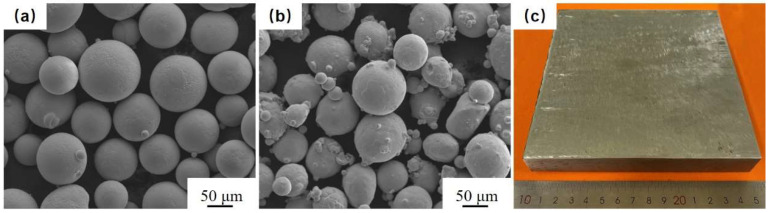
Experimental material: (**a**) Ni60B powder, (**b**) Ni powder, (**c**) 38CrMoAl substrate.

The powder material for the fusion cladding layer was selected from Ni60B, a nickel-based alloy, with a powder particle size of 45–105 μm, and the powder material for doping the transition layer was selected from pure nickel with a powder particle size of 45–105 μm (Figure 2). The substrate was selected from 38CrMoAl steel with dimensions of 150 × 150 × 20 mm (Figure 2), and the main compositions of the powder and substrate are shown in Table 1. Prior to the experiment, the substrate was sanded with 400# sandpaper to remove surface contaminants, residual stains were wiped off with a degreasing cotton pad dipped in anhydrous ethanol, and the powder was placed in a drying oven at 120 °C for 4 h.

### 2.2. Methods of Experimental and Test Analysis

**Figure 3:**
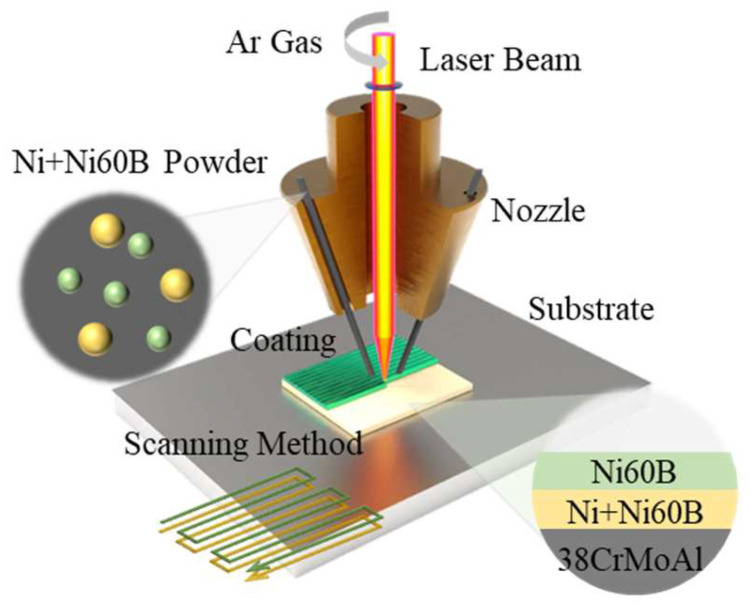
Experimental schematic.

As shown in Figure 3, in order to investigate the effect of process parameters on the cracking of Ni60B coatings, the fusion cladding experiments were initially carried out using a single-pass, single-layer experiment (repeat each experimental parameter three times to reduce error interference). Among them, the laser power is in the range of 1000~1800 W, the scanning speed is in the range of 2~10 mm/s, and the coating thickness (matching the powder feeding rate) is selected in the interval of 0.2~1.0 mm. Subsequently, single-layer multi-pass fabrication experiments were carried out to observe the cracking of Ni60B coatings and ultimately determine the process parameters for subsequent crack analysis and inhibition experiments by selecting representative combinations of process parameters (laser power, 1600 W; scanning speed, 6 mm/s; high coating thickness, 0.6 mm). First, we prepared Ni containing transition coatings on 38CrMoAl substrates and waited for the substrates to be lowered to room temperature before depositing Ni60B coatings on the surface of the transition layer. The transition layer schemes are shown in Table 2. This results in good surface morphology and crack-free samples by avoiding the effects of heat buildup on the coating preparation. For macroscopic crack detection during the experiment, the surface of the coating was colored using the DPT-5 surface coloring probe to observe cracks in the fused cladding.

The surfaces of the collected samples were ground and polished, and the physical phase species were determined using an Empyrean X-ray diffractometer from Panacor, Almelo, The Netherlands. A Hitachi SU5000 scanning electron microscope (Tokyo, Japan) was used to photograph the crack section in secondary electron mode and the longitudinal section near the crack in backscatter mode, and X-ray spectroscopy was used to determine the elemental composition of the different objects. For the intercepted sample longitudinal section after grinding and polishing, the HV-1000A Vickers hardness tester (Yingjianda Instruments Co., Chongqing, China) was used to measure the hardness of the fused cladding layer under different transition programs using a load of 1.961 N, a loading time of 15 s, the first sample point from the fused cladding layer at a longitudinal distance of 0.05 mm (from the fused cladding layer to the direction of the substrate), and the longitudinal distance of each tsetpoint for the 0.05 mm; each sample piece took seven tset points. Each sample point has a longitudinal distance of 0.05 mm.

## 3. Results and Discussion

### 3.1. Analysis of Cracking Behavior of Ni60B Coating

#### 3.1.1. Influence of Process Parameters on the Cracking Behavior of Coatings

**Figure 4:**
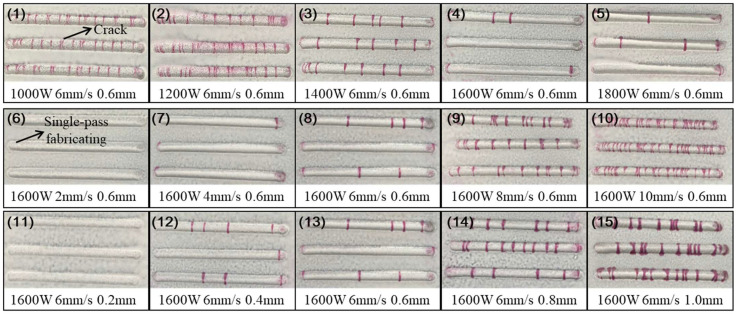
Penetrant inspection results of coating.

This section involved the investigation of the effect of process parameters on the cracking susceptibility of Ni60B coatings using single-pass single-layer experiments. A single-factor experiment was used to select representative process parameters to observe coating cracking. Specific combinations of process parameters are shown in Table 3. According to the parameters selected to carry out the laser melting fabrication experiments, the preparation of 50 mm single-pass single-layer specimens, the crack detection of each specimens, and the results of flaw detection by colouring are shown in Figure 4.

**Figure 5:**
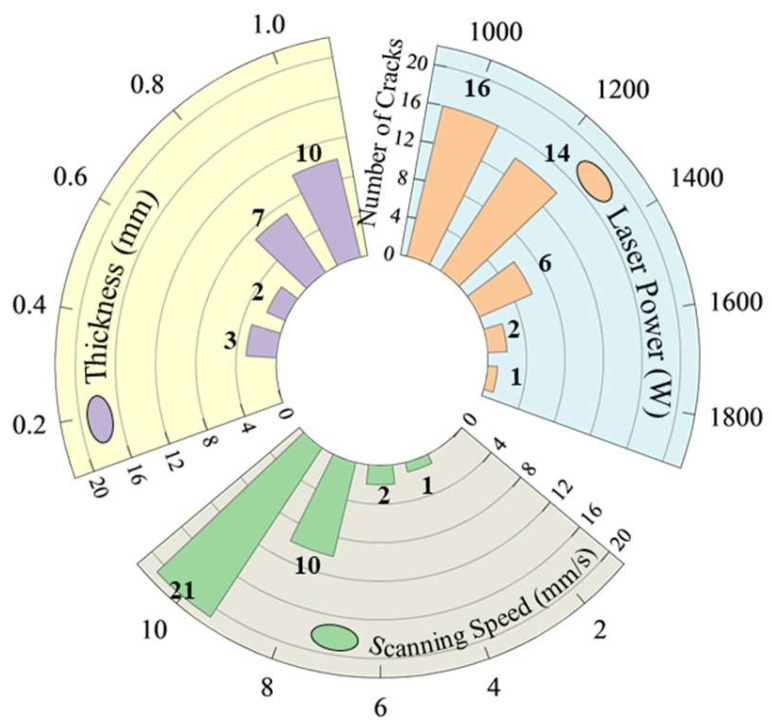
Number of cracks statistics.

Through the coloring flaw map, statistics were made on the direction and number of cracks in the coating. It was observed that, under single-pass single-layer conditions, the cracking direction is perpendicular to the scanning speed. As for the number of cracks, Figure 4 shows that it varies with different process parameters. A statistical analysis of the average number of cracks for samples with varying process parameters is presented in Figure 5. The results indicate that, while maintaining a constant layer thickness, an increase in laser power leads to higher heat input, resulting in a significant reduction in the number of cracks. Conversely, as the scanning speed increases, the heat input decreases, causing a marked increase in the number of cracks. This is because increased heat input and prolonged molten pool duration improve fluidity, reduce the temperature gradient, slow the solidification rate, lower residual stresses, and thus decrease crack susceptibility in the coating [26]. When laser power and scanning speed are kept constant, but layer thickness is varied, an increase in layer thickness leads to more pronounced cracking. This occurs because thicker coatings require more heat to melt the powder, reducing the melt time and fluidity, increasing the temperature gradient, and elevating residual stresses, all of which heighten the coating’s susceptibility to cracking [27].

**Figure 6:**
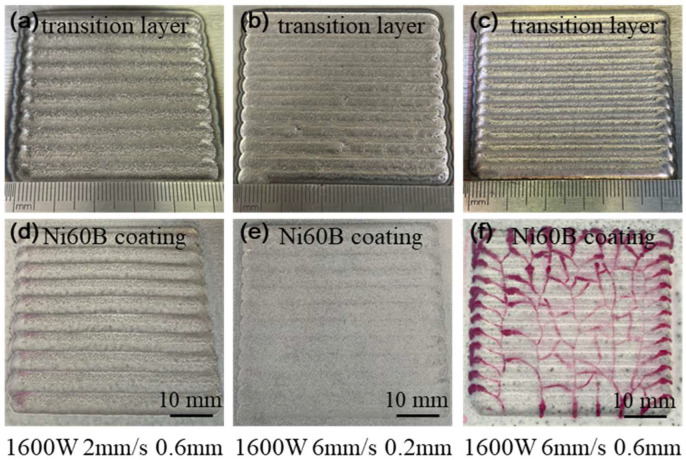
Single-layer multi-beads coatings and penetrant inspection result: (**a**,**d**) P = 1600, V = 2 mm/s, and H = 0.6 mm. (**b**,**e**) P = 1600 W, V = 6 mm/s, and H = 0.2 mm. (**c**,**f**) P = 1600, V = 6 mm/s, and H = 0.6 mm.

Based on the observed cracking behavior, under the experimental conditions used in this study, when the laser power is below 1600 W, the scanning speed exceeds 6 mm/s, and the coating thickness is greater than 0.6 mm, Ni60B single-pass, single-layer samples exhibit high sensitivity to cracking, making these conditions unsuitable for the laser melting and fabrication of Ni60B coatings. To address this, the process parameters listed in Table 4 were used to produce a single layer with multiple passes, and the results of surface analysis using a color probe are shown in Figure 6. As seen in Figure 6a,b, lower deposition rates and thinner deposition layers can result in crack-free coatings, but these conditions are impractical for real-world applications due to their low deposition efficiency and insufficient layer thickness. In contrast, the process parameters of P = 1600 W, V = 6 mm/s, and a coating thickness of 0.6 mm (corresponding to a powder feed rate of 5.2 g/min) strike a balance between deposition efficiency and coating thickness. However, while this combination results in fewer cracks during single-pass deposition, it still leads to more severe cracking in multi-pass overlapping deposits, as shown in Figure 6c.

#### 3.1.2. Analysis of Coating Cracking Mechanism

**Figure 7:**
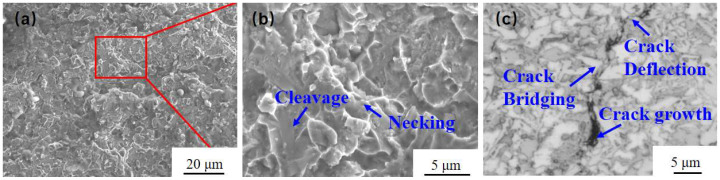
Fracture morphology and micro-crack morphology diagram: (**a**) macroscopic fracture morphology, (**b**) high-magnification fracture morphology, and (**c**) micro-crack morphology.

Figure 7a shows the macroscopic morphology of the fracture surface, while Figure 7b illustrates the microscopic morphology of the cladding layer. Figure 7c displays the microstructure of the fusion cladding in a cross-section perpendicular to the direction of the crack. The fracture surface in Figure 7a exhibits distinct undulations, with parts of the surface appearing angular with a metallic luster, as well as flat, stepped areas. The angular, lustrous corners show clear necking characteristics, indicating high toughness in these regions, and some evidence of plastic deformation at the point of fracture, which helps inhibit crack propagation. In contrast, the stepped areas are indicative of brittle fracture, caused by the hardness and brittleness of the grains at those points. These grains crack easily and produce smooth, flat, brittle fracture surfaces when subjected to relatively weak forces.

The cross-sectional morphology of the crack reveals the characteristics of a perforation crack that propagates along the gray phase. These cracks are coarse and deep, corresponding to the brittle fracture areas seen in Figure 7a. When the crack passes through the white phase, it becomes narrower and shallower, indicating a suppressed state due to the improved toughness of the material in this region, which aligns with the necking features previously described.

**Figure 8:**
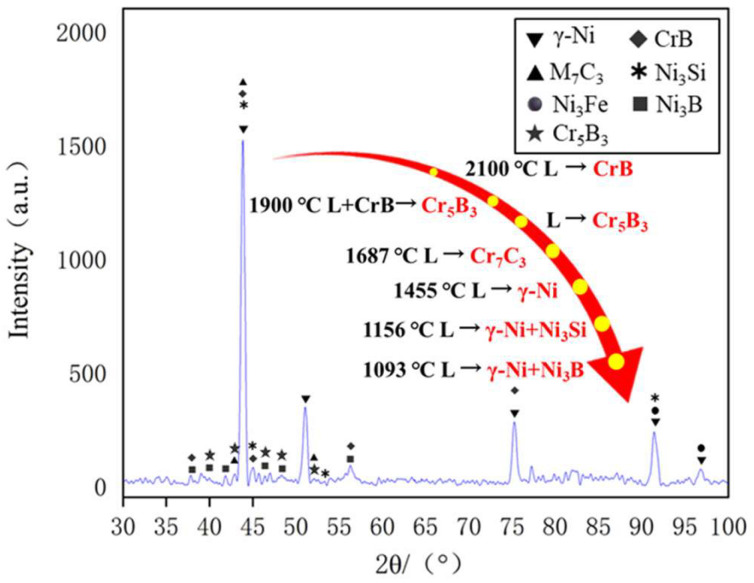
XRD of Ni60B coatings.

The Ni60B sample was subjected to X-ray diffraction (XRD) spectroscopy as shown in Figure 8. Combined with existing research, the main phases of Ni60B coatings can be deduced from comparative analyses to be γ-Ni, M_7_C_3_ (M=Cr, Fe and Ni), Ni_3_B, Ni_3_Fe, Ni_3_Si, CrB, and Cr_5_B_3_ [12,25,28]. Hard phases such as Cr-borides and carbides are first formed in the cladding layer, and Ni dendrites and Ni-Cr-B eutectics grow with the decrease in molten pool temperature [29]. As shown in the references, CrB, Cr_5_B_3,_ and Cr_7_C_3_ hard phases are first precipitated in the molten pool [29,30], where Cr_5_B_3_ can be precipitated both directly from the molten pool and indirectly through inclusion reactions. Subsequently, γ-Ni solid solution is formed in the molten pool, and finally two eutectic microstructures, Ni-Ni_3_Si and Ni-Ni_3_B, are produced by eutectic reaction.

**Figure 9:**
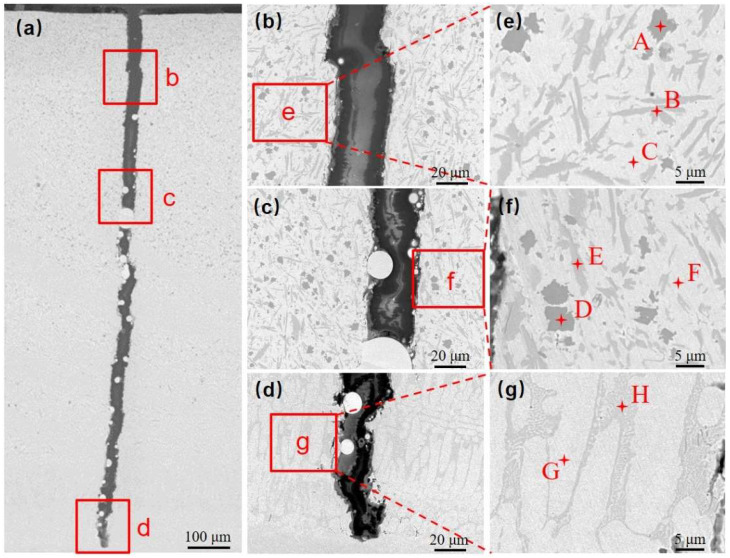
Phase and EDS analysis of Ni60B coating: (**a**) macro phases, (**b**) Microstructure of the upper part of the coating, (**c**) Microstructure of the middle part of the coating, (**d**) Microstructure of the upper part of the coating, (**e**) EDS analysis of the middle part of the coating, (**f**) EDS analysis of the middle part of the coating, (**g**) EDS analysis of the bottom part of the coating.

A longitudinal section perpendicular to the crack location was taken and the microstructure of the Ni60B coating was observed by scanning electron microscopy (SEM) at the top, middle, and near the bottom of the Ni60B coating, as shown in Figure 9a–d. And the EDS test results are shown in Table 5. The upper and middle parts of the coating are mainly composed of a black solid phase, a gray irregular stripe phase, a gray butterfly phase, and a white basal phase, while the lower part of the coating is mainly composed of white and gray basal phases, and the black, and gray precipitate phases appearing in the upper and middle parts of the coating are not seen. The main constituents of the massive black phase (point A and point D) are Cr and B, and the atomic ratio is close to 1:1, so the black precipitation phase is CrB. The irregular bar phase (point B) is dominated by Cr and C as the main constituent elements but also contains high amounts of Ni and Fe. The reason is that Ni (0.352 nm), Cr (0.288 nm), and Fe (0.287 nm) atoms have very similar atomic radii, and they are very easy to exchange with each other [31], and it can be deduced that the gray phase is M_7_C_3_ (M=Cr, Fe and Ni). The main constituents of the gray butterfly phase (point E) are Cr and B. Combined with the atomic ratio, it can be deduced that this gray phase is Cr_5_B_3_. The white basal phase (point C and point F) is mainly dominated by Ni and Fe elements, and this basal phase is a γ-Ni solid solution. As a result of the laser cladding process, some of the elements in the substrate are diluted into the coating, making the bottom of the coating rich in Fe elements. Excessive Fe elemental content inhibits the precipitation of hard phases such as incipient borides, resulting in the presence of only two basal phases, white and gray [32]. Combining Fe and Ni elements with good mutual solubility [31], the white basal phase is the γ-Ni solid solution. And the gray basal phase is the Ni-B-Si eutectic microstructure composed of Ni_3_Fe, Ni_3_B, and Ni_3_Si. The high content of element B in the molten pool initially develops a hard boride phase with a high melting point during solidification [12]. The remaining B element is not enough to form a large amount of the Ni-B-Si eutectic microstructure, which in turn leads to the absence of obvious Ni-B-Si eutectic in the top and middle parts of the Ni60B coating.

**Figure 10:**
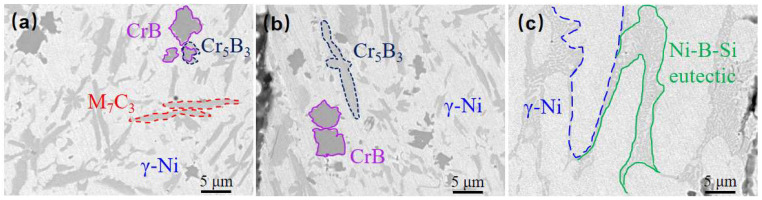
Phase of Ni60B coating: (**a**) upper part of coating, (**b**) middle part of coating, and (**c**) bottom part of coating.

In summary, the basal phase of the Ni60B coating consists of γ-Ni and Ni-B-Si eutectic composed of Ni_3_B, FeNi_3_, and Ni_3_Si, and the hard precipitation phase consists of M_7_C_3_ (M=Cr, Fe, Ni), CrB, and Cr_5_B_3_ (Figure 10). The top and middle part of the Ni60B coating contains a large number of unevenly distributed, large-sized hard phases, which increase the hardness of the coating while also increasing its brittleness.

Laser cladding is a rapid heating and cooling process. During solidification, the remaining liquid metal may not fill the gaps caused by the contraction of the solidifying metal in time. As the solid-state cooling process continues, the surrounding colder substrate binds the metal, leading to the formation of significant residual stress. This residual internal stress is often not well released. When the strain caused by this residual stress exceeds the material’s strain limit, cracks may form. These cracks are primarily the result of thermal stress. The thermal stress equation is as follows [33]:

(1) $$\sigma_{T}=\frac{E_{c}E_{s}t_{s}\left(\alpha_{c}-\alpha_{s}\right)∆T}{\left(1-v\right)\left(E_{s}t_{s}+E_{c}t_{c}\right)}$$

where $\sigma_{T}$ is the residual thermal stress of the coating, *E* is the modulus of elasticity of the material, *t* is the thickness, *α* is the coefficient of the thermal expansion of the material, ∆*T* is the temperature difference in the coating (difference between the fabricating temperature and the substrate temperature), and *v* is the Poisson’s ratio of the fusion-coated material, where the subscript *c* refers to the coating material and the subscript s refers to the substrate material. From the above Equation (1), it can be seen that the thermal stress to which the coating is subjected is mainly influenced by the temperature difference between the fused cladding layer and the substrate, the difference between the coefficients of the thermal expansion of the fused cladding layer material and the substrate material, and the thickness of the fused cladding layer, among other factors. The coefficient of the thermal expansion of nickel-based coatings is (13.4~16.8) × 10^−6^ K^−1^ and that of carbon steel is (11.7~13.9) × 10^−6^ K^−1^. When $\sigma_{T}$ is positive, the coating is subjected to tensile stress and the maximum equivalent stress occurs at the top position of the coating. A high-power laser beam irradiates the surface of the metal material, and the laser energy is absorbed by the metal and converted into heat. Short-term heat is not conducted to the interior of the molten cladding layer, most of the heat energy is collected at the surface of the molten cladding area and forms thermal stresses, which become an important cause of crack initiation [26,34].

### 3.2. Effect of Ni Transition Layer on Inhibition of Coating Cracking

The analysis of the cracking mechanism indicates that reducing the difference in thermal expansion coefficients between the coating and substrate materials can directly decrease thermal stress during the deposition process, thereby inhibiting cracking. To achieve this, a suppression scheme involving a transition layer of Ni elements between the substrate and the fusion cladding layer is proposed. On one hand, the thermal expansion coefficient of Ni is 12.8 × 10^−6^ K^−1^, which falls between that of Ni60B and 38CrMoAl steel. By incorporating a transition layer containing Ni, the difference in thermal expansion coefficients between the substrate and the Ni60B coating can be effectively minimized. On the other hand, during the cladding of Ni60B coatings, the laser remelting effect facilitates the diffusion of Ni from the transition layer into the cladding layer, enhancing the toughness of the coating. Therefore, the addition of a nickel powder transition layer between the substrate and the Ni60B coating is expected to significantly aid in crack suppression.

#### 3.2.1. Effect of Ni-Doped Transition Layer on Coating Cracking Inhibition

**Figure 11:**
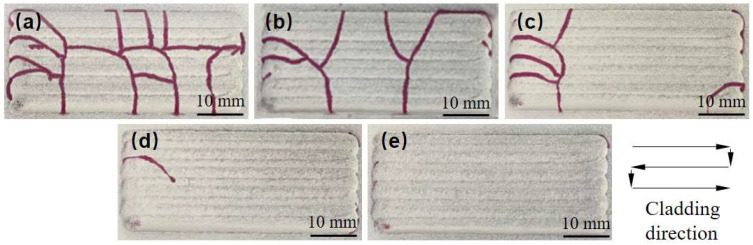
Penetration inspection results of different transition schemes (Red lines are cracks showing colour): (**a**) transition scheme a. (**b**) Transition scheme b. (**c**) Transition plan c. (**d**) Transition plan d. (**e**) Transition plan e.

To investigate the inhibition effect of the cracking of Ni60B coatings using different transition schemes as shown in Table 2, the fabricating morphology and flaw detection results are shown in Figure 11. The inhibition method using the addition of a transition layer is more effective than Ni doping for the overall inhibition of coating cracking. The reason for this is that the transition method combines the effects of improving the toughness of the coating and reducing the residual thermal stresses in the coating. This is because the transition method effectively combines the benefits of enhancing the toughness of the coating and reducing the residual thermal stresses within it. The susceptibility of the coating to cracking decreases significantly as the Ni content in the transition layer increases. When the mass fraction of the Ni elements doping in the transition layer reaches 100% (transition scheme e), crack-free Ni60B coating samples can be achieved. Examining the cracking locations in Figure 11 reveals that the cracks are predominantly located on both sides of the coating. This phenomenon occurs due to the “S” type cladding route, where the longer dwell time of the laser head at the side transition points allows for the greater absorption of the powder. The increased dwell time of the laser nozzle leads toa large waviness in the position of the coating on both sides. When laser fusion cladding is performed along the transition layer path, the susceptibility to cracking increases with the coating thickness at both side locations. This is accompanied by a more complex stress distribution, which in turn makes the coating more susceptible to cracking at both side locations.

#### 3.2.2. Mechanism of Cracking Inhibition of Coatings by Ni-Doped Transition Layers

**Figure 12:**
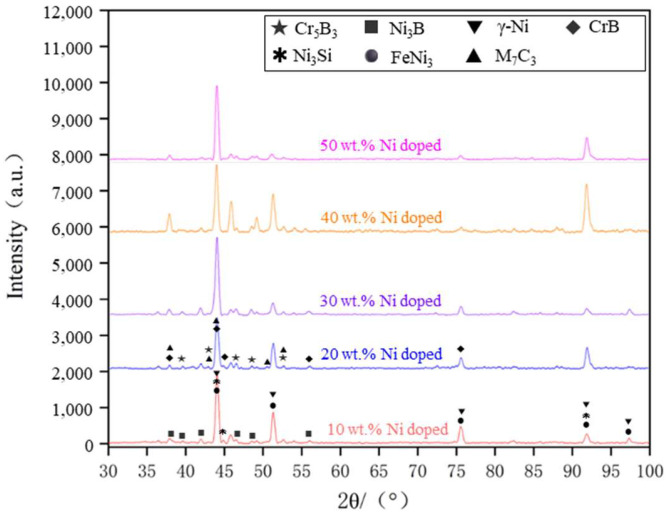
XRD patterns of different Ni doping qualities.

Figure 12 presents the XRD spectra of the coatings with varying levels of Ni doping. By analyzing the X-ray diffraction (XRD) spectra under different conditions of Ni doping, it is evident that the main phases of the coating consist of γ-Ni, M_7_C_3_ (M=Cr, Fe, Ni), Ni_3_B, Ni_3_Fe, Ni_3_Si, CrB, and Cr_5_B_3_. The positions of the diffraction peaks for the coating phases under various transition conditions remain largely consistent, with differences observed only in the intensity of some peaks. An analysis of the XRD spectra suggests that the main phase composition of the cladding layer is similar across different transition schemes, indicating that the transition method effectively preserves the material’s inherent phase properties.

**Figure 13:**
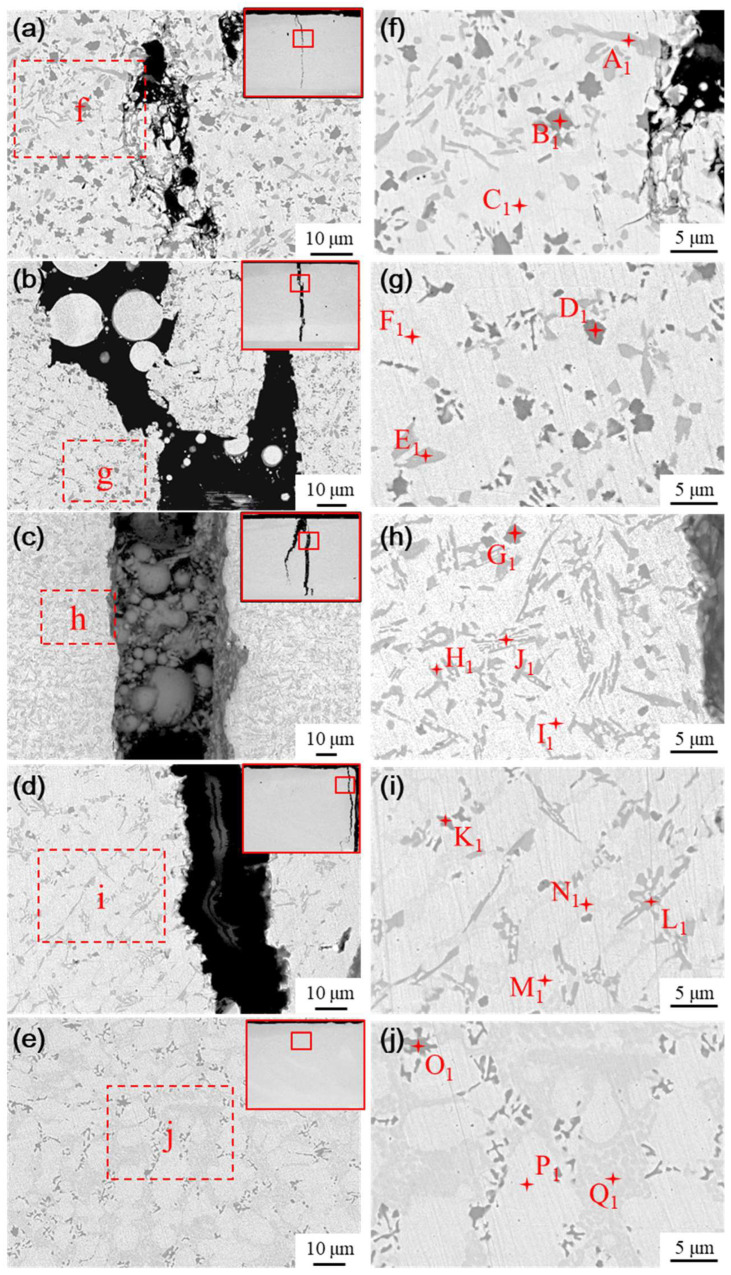
Physical phase and EDS analysis of cladding layer with different transition schemes: (**a**) Microstructure of scheme a, (**b**) Microstructure of scheme b, (**c**) Microstructure of scheme c, (**d**) Microstructure of scheme d, and (**e**) Microstructure of scheme e, (**f**) Analysis of scheme a, (**g**) Analysis of scheme b, (**h**) Analysis of scheme c, (**i**) Analysis of scheme d, (**j**) Analysis of scheme e.

The microstructure of the fused cladding layer with different transition schemes was examined using SEM, as shown in Figure 13. As a result of laser remelting, some Ni elements from the transition layer diffuse into the cladding layer, thereby influencing its physical phases. As the Ni content infiltrating the molten cladding increases, both the quantity and size of the black and gray precipitated phases decrease, while the proportion of ductile phases in the substrate increases. The microstructure evolves from a single white basal phase to a coexistence of white and gray phases, along with the emergence of a new phase in Figure 13b, characterized by clusters of gray precipitated phases interspersed within the white basal phase.

Energy Dispersive Spectroscopy (EDS) was conducted to analyze the elemental composition of the typical physical phases in the medium fusion cladding layer, with the results presented in Table 6. The physical phases of the fused cladding layers prepared under transition schemes a and b are similar to the microstructure of the pure Ni60B coating. This similarity arises from the lower content of Ni elements infiltrating the fused cladding layer, which helps preserve the physical phase characteristics of the original Ni60B coating. The main constituent elements of the massive black phase (point B_1_ and point D_1_) are Cr and B, and the atomic ratio is close to 1:1, so the black precipitation phase is CrB. The primary constituent elements of the irregular strip phase (points A_1_ and E_1_) are mainly Cr and C, along with higher concentrations of Ni and Fe. This analysis is significant because the atomic radii of Ni (0.352 nm), Cr (0.288 nm), and Fe (0.287 nm) are extremely similar, facilitating their interchangeability [31]. The combined atomic ratios of these three main metal elements, along with carbon, approximate to 7:2, indicating that the gray phase is M_7_C_3_ (where M=Cr, Fe, and Ni). The borides and carbides of Cr have a larger ΔGf than the corresponding compounds of Fe and Ni, so Ni60B coatings will preferentially form borides and carbides of Cr. During solidification, chromium borides precipitate first, followed by the nucleation of chromium carbides that attach to the initially precipitated borides. As the chromium carbide grows, it ultimately forms irregular radial structures [29]. The white basal ductile phase (points C_1_ and F_1_) consists of a γ-Ni solid solution rich in Fe and a small amount of Si. The combined Fe and Ni elements exhibit good mutual solubility [31]. The white basal phase is a γ-Ni solid solution, while the gray basal phase comprises a Ni-B-Si eutectic structure formed from Ni_3_Fe, Ni_3_B, and Ni_3_Si.

As shown in Figure 13c, when the mass fraction of nickel doping in the transition layer reaches 60% (scheme c), the physical phase of the coating produces more obvious changes compared with the physical phase characteristics of the pure Ni60B coating. The black massive hard phase (point G_1_), the gray irregularly striated phase (point J_1_), and the white basal phase (point I_1_) are consistent with previous observations, identified as CrB, M_7_C_3_, and a γ-Ni solid solution, respectively. Additionally, a gray precipitated phase interspersed with the white basal phase appears as a flower cluster phase (point H_1_), primarily composed of B, Cr, Fe, and Ni. This physical phase is presumed to be a eutectic microstructure consisting of Cr_2_B + γ-Ni, based on the existing literature [30,35]. This assumption is supported by the fact that after the precipitation of hard phases like CrB and M_7_C_3_, the remaining Ni content is insufficient to form a substantial number of eutectic crystals comprising Ni_3_B, Ni_3_Si, and Ni_3_Fe. The symmetric equivalence transition occurs at 1222 °C, allowing the eutectic microstructure to precipitate directly from the liquid phase. The main physical phases of transition scheme c include M_7_C_3_, a γ-Ni solid solution, a eutectic composed of Cr_2_B and Ni, and a small amount of fine CrB.

**Figure 14:**
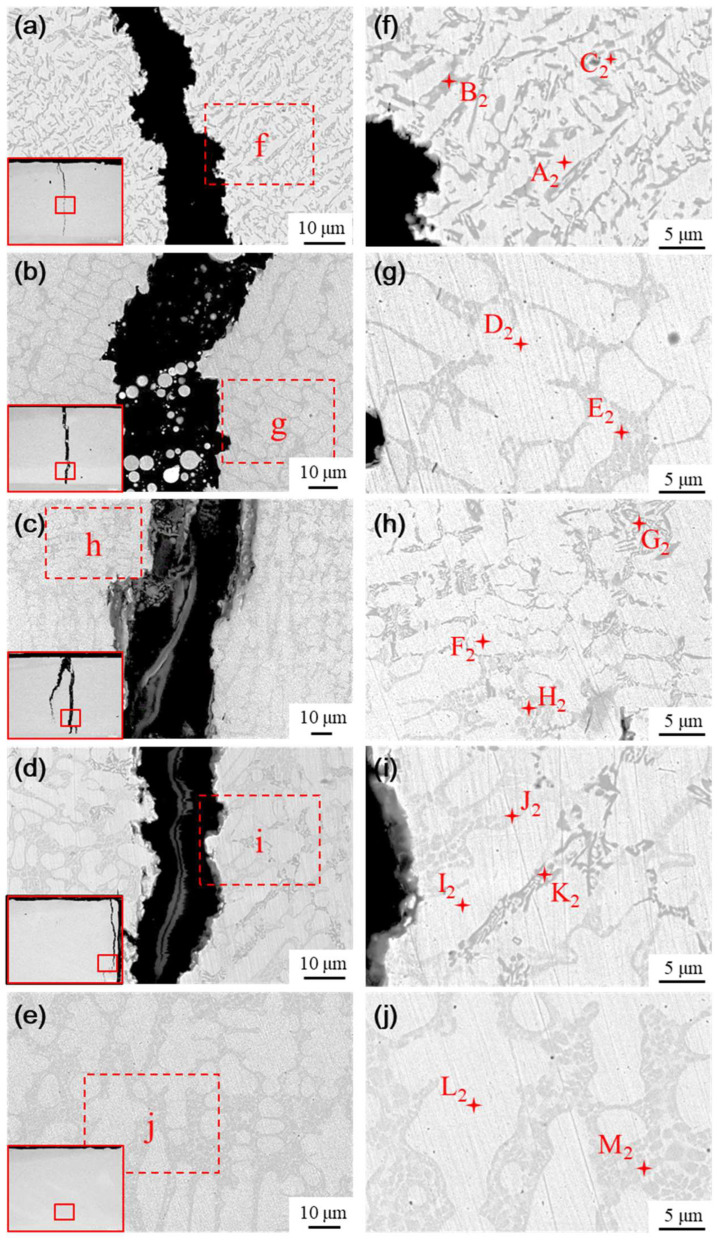
Physical phase and EDS analysis of transition layer with different transition schemes: (**a**) Microstructure of scheme a, (**b**) Microstructure of scheme b, (**c**) Microstructure of scheme c, (**d**) Microstructure of scheme d, and (**e**) Microstructure of scheme e, (**f**) Analysis of scheme a, (**g**) Analysis of scheme b, (**h**) Analysis of scheme c, (**i**) Analysis of scheme d, (**j**) Analysis of scheme e.

With the further enhancement of nickel doping in the transition layer, as shown in Figure 13d and Figure 14e, the percentages of the lumpy black phase and the gray irregularly striped phase decreased significantly. The basal phase transitioned from a purely white phase to a coexistence of both white phase (dots M_1_ and P_1_) and gray phase (dots N_1_ and Q_1_). Notably, the gray basal phase contains a higher concentration of B elements compared to the white basal phase. This change can be attributed to the infiltration of a substantial amount of nickel into the cladding layer, resulting in significant alterations in the elemental composition during the solidification process. Consequently, a large quantity of Ni interacts with B, Si, and Fe elements to form a eutectic structure composed of Ni_3_B, Ni_3_Si, and Ni_3_Fe, among others. Therefore, the main physical phases under the aforementioned transition schemes consist of a γ-Ni solid solution, Ni-B-Si eutectic, and a small amount of boride and carbide hard phases. In summary, as the content of Ni elements infiltrating the transition layer increases, both the number and size of the hard phases in the fusion-coated layer decrease, while the size and proportion of the γ-Ni tough phase increases, which enhances the toughness of the coating to some extent.

The microstructure of the transition layer is illustrated in Figure 14. The physical phase characteristics in the transition layer significantly differ from those of the fused cladding layer; specifically, the black and gray precipitated hard phases nearly disappear as the content of the Ni element increases. The predominant phases in the transition layer are primarily white and gray basal phases, accompanied by a small number of gray precipitated phases and flower cluster-like phases interspersed among the white basal phases. The elemental composition of the typical phases in the transition layer was analyzed using EDS, with the results presented in Table 7. The phases formed in the transition layer under various transition schemes are similar, primarily consisting of Cr_2_B + γ-Ni eutectic structures (points B_2_, E_2_, and J_2_), with flower cluster-like phases situated between the gray precipitated phases and the white basal phases, as well as gray basal phases formed by Ni-B-Si eutectic structures (points D_2_, F_2_, I_2_, and L_2_), and γ-Ni solid solutions with white basal phases (points A_2_, C_2_, G_2_, H_2_, and K_2_).

As nickel doping in the transition layer increases, the number of hard phases decreases or even vanishes, while the size and proportion of the base toughness phase increase, enhancing the toughness of the transition layer. The high concentration of Ni elements in the transition layer inhibits the precipitation of hard phases to some extent, leading to the formation of γ-Ni solid solutions and Ni-B-Si eutectic structures, along with a small amount of Cr_2_B + γ-Ni eutectic phases.

#### 3.2.3. Effect of Different Transition Schemes on the Microhardness of Coatings

**Figure 15:**
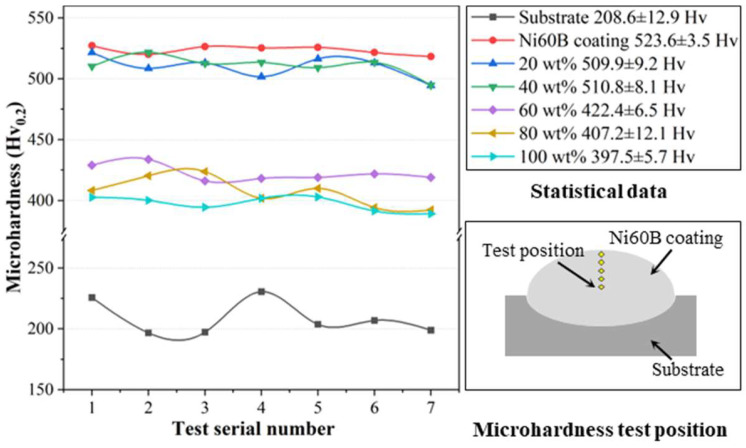
Microhardness of different transition schemes.

The microhardness curves are illustrated in Figure 15, demonstrating that the microhardness values of the fused cladding layer under each transition scheme are relatively consistent, indicating good uniformity in the fused cladding layer. The average microhardness of the Ni60B coating fabricated directly on the substrate reached 523.6 ± 3.5 HV_0.2_, which is a 2.52-fold increase compared to the 208.6 ± 12.9 HV_0.2_ of the substrate.

The microhardness of the fused cladding layer gradually decreases with the increasing of Ni element doping, which weakens the diffuse strengthening effect of the low-microhardness phases in the coating [36,37]. However, a higher concentration of Cr atoms is solidly dissolved in the γ-Ni solid solution, enhancing its microhardness properties, while the Ni-B-Si eutectic also maintains good microhardness characteristics [28]. These two factors contribute to the coatings with higher Ni doping still exhibiting commendable microhardness. Based on the differences in microhardness values, the coatings can be categorized into two groups corresponding to different transition schemes. The first group includes transition schemes a and b, with microhardness values similar to the original Ni60B coatings. The second group comprises transition schemes c, d, and e, which exhibit significantly lower microhardness than the original coatings. In the first group, the microhardness values reached 509.9 ± 3.5 HV_0.2_, primarily due to the presence of borides and carbides in the fusion cladding, which enhance microhardness through a diffuse strengthening effect. In the second group, as the Ni content increased from 60 wt.% to 100 wt.%, the microhardness decreased from 422.4 ± 6.5 HV_0.2_ to 397.5 ± 5.7 HV_0.2_. Excessive Ni doping leads to this reduction. However, the microhardness remains significantly higher than that of the substrate (208.6 HV_0.2_). Although the amount and size of hard phases in the coating are reduced, the solid solution strengthening effect is enhanced by the increased Cr elements in the γ-Ni solid solution. Furthermore, the eutectic structures comprising Cr_2_B + γ-Ni and Ni-B-Si also contribute to higher microhardness [28].

In summary, while introducing a transition layer with varying Ni content suppresses cracking in the coating, excessive Ni from the transition layer impacts the microhardness of the fusion cladding layer. Nevertheless, experiments indicate that within a certain range of Ni content in the transition layer, the effect on coating microhardness properties is minimal.

## 4. Discussion

In this study, the effects of process parameters on the crack sensitivity of Ni60B coatings were summarized. Methods for crack suppression were analyzed, including the doping of Ni elements and the application of a transition layer. The effectiveness of these methods in suppressing cracks and the impact on microstructure properties were compared. The following conclusions were drawn:

1. The crack sensitivity of the Ni60B coating decreases as laser power increases but rises with higher scanning speeds and increased coating thickness. The main phases of the coating include γ-Ni, M_7_C_3_ (M=Cr, Fe, and Ni), Ni_3_B, FeNi_3_, Ni_3_Si, CrB, and Cr_5_B_3_. The transition layer containing Ni elements did not significantly alter the phase composition of the cladding coating.
2. Increasing the amount of Ni powderdoping reduces the crack sensitivity of the coating, increases the proportion of tough phases, and decreases the amount of hard precipitate phases. A fully Ni transition layer (100 wt.% Ni) allows for the crack-free formation of the coating. As the Ni elements in the transition layer increase, the proportion of tough phases in the coating also increases, while both the size and quantity of the hard precipitate phases decrease.
3. The average microhardness of the Ni60B coating directly formed on the substrate reached 523.6 ± 3.5 HV_0.2_, which is 2.5 times higher than the 208.6 ± 12.9 HV_0.2_ of the substrate. After adding the transition layer, the microhardness decreased with increasing Ni elements in the transition layer; when Ni < 40 wt.%, the top Ni60B coating microhardness reached 509.9 ± 3.5 HV_0.2_. However, when Ni > 60 wt.%, the microhardness of the top Ni60B coating decreased from 422.4 ± 6.5 HV_0.2_ to 397.5 ± 5.7 HV_0.2_, showing an improvement of over 91% in microhardness compared to the substrate.

## Figures and Tables

**Table 1:** Composition and content of Ni60B powder and 38CrMoAl substrate (wt.%).

Materials	Ni	Fe	Cr	Si	B	C	Al	Mo	Cu
Ni60B	Bal.	<15	14~19	3.5~5.0	3.0~4.5	0.5~1.0	-	-	-
38CrMoAl	≤0.3	Bal.	1.4~1.6	0.2~0.4	-	0.3~0.4	0.7~1.1	0.1~0.2	≤0.3

**Table 2:** Transition schemes.

No.	Constituents of the Transition Layer	Composition of the Cladding Layer	Crack Condition
1	20 wt.% Ni + 80 wt.% Ni60B	100 wt.% Ni60B	Y
2	40 wt.% Ni + 60 wt.% Ni60B	100 wt.% Ni60B	Y
3	60 wt.% Ni + 40 wt.% Ni60B	100 wt.% Ni60B	Y
4	80 wt.% Ni + 20 wt.% Ni60B	100 wt.% Ni60B	Y
5	100 wt.% Ni + 0 wt.% Ni60B	100 wt.% Ni60B	N

**Table 3:** Process parameter table of single-pass single-layer experiments.

No.	Laser Power (W)	Scanning Speed (mm/s)	Thickness (mm)	Average Number of Cracks
1	1000	6	0.6	16
2	1200	6	0.6	14
3	1400	6	0.6	6
4	1600	6	0.6	2
5	1800	6	0.6	1
6	1600	2	0.6	0
7	1600	4	0.6	1
8	1600	6	0.6	2
9	1600	8	0.6	10
10	1600	10	0.6	21
11	1600	6	0.2	0
12	1600	6	0.4	3
13	1600	6	0.6	2
14	1600	6	0.8	7
15	1600	6	1.0	10

**Table 4:** Process parameter table of coating experiments.

No.	Laser Power (W)	Scanning Speed (mm/s)	Thickness (mm)
a	1600	2	0.6
b	1600	6	0.2
c	1600	6	0.6

**Table 5:** Elemental composition table of main phases in Figure 9 (at.%).

Point	B	C	Si	Cr	Fe	Ni
A	40.24	5.88	0.12	45.03	7.21	1.52
B	5.81	21.16	1.18	35.53	22.98	13.34
C	5.10	10.57	6.35	2.90	19.67	55.41
D	40.49	5.91	0.10	46.06	6.19	1.24
E	23.99	7.72	0.26	33.82	25.27	8.94
F	3.74	10.56	7.42	2.74	17.99	57.55
G	0.00	8.56	1.45	3.26	75.33	9.96
H	13.35	10.36	0.97	6.93	59.45	8.95

**Table 6:** Elemental composition table of main phases in Figure 13 (at.%).

Point	B	C	Si	Cr	Fe	Ni
A_1_	40.11	5.01	0.18	45.69	6.71	2.3
B_1_	6.25	20.84	1.02	34.5	22.54	14.85
C_1_	8.94	8.91	8.92	2.64	15.13	55.45
D_1_	38.55	6.03	0.59	43.31	7	4.52
E_1_	5.1	19.68	0.41	32.01	24.78	18. 02
F_1_	-	10.06	3.45	6.82	27.31	52.15
G_1_	36.37	5.84	2.16	37.11	8.21	10.31
H_1_	8.52	14.39	2.71	22.03	27.31	26.04
I_1_	2.33	9.72	6.92	3.55	21.13	56.35
J_1_	13.94	10.31	2.71	15.68	26.7	30.66
K_1_	40.45	6.28	0.24	41.48	9.11	2.45
L_1_	6.26	19.21	0.74	25.16	26.37	23.26
M_1_	-	11.24	2.53	5.92	27.88	52.44
N_1_	16.92	9.66	4.67	7.60	14.34	46.80
O_1_	41.63	6.86	0.42	36.15	9.27	5.67
P_1_	-	10.53	3.13	6.79	27.18	52.37
Q_1_	10.29	11.12	2.37	6.37	20.82	49.04

**Table 7:** Elemental composition table of main phases (at.%).

Point	B	C	Si	Cr	Fe	Ni
A_2_	-	10.31	3.89	3.45	35.8	46.55
B_2_	19.81	8.04	1.66	15.42	32.72	22.35
C_2_	7.54	8.85	2.61	5.91	31.38	43.71
D_2_	-	12.57	1.73	3.54	36.92	45.24
E_2_	10.28	10.18	0.64	5.96	33.71	39.23
F_2_	-	10.25	2.15	5.27	43.96	38.36
G_2_	14.82	9.37	3.05	8.77	29.84	34.16
H_2_	5.54	10.94	3.59	5.59	33.92	40.42
I_2_	-	10.75	2.33	6.41	31.69	48.82
J_2_	9.83	10.65	2.98	7.36	22.38	46.8
K_2_	17.14	9.83	2.45	11.16	26.3	33.12
L_2_	-	10.25	2.43	6.33	28.36	52.63
M_2_	13.57	9.68	0.83	9.8	22.52	43.60

## Data Availability

The original contributions presented in this study are included in this article, and further inquiries can be directed to the corresponding author.
